# Patterns of emergency care utilization among First Nations residents of Wood Buffalo, Alberta, during public health crises: An interrupted time series analysis

**DOI:** 10.17269/s41997-026-01238-2

**Published:** 2026-07-23

**Authors:** Erynne Sjoblom, Barbara S. E. Verstraeten, Stephanie Montesanti

**Affiliations:** School of Public Health, Dianne and Irving Kipnes Health Research Academy, University of Alberta, Edmonton, AB Canada

**Keywords:** Indigenous Peoples, Interrupted time series analysis, Routinely collected health data, Wildfires, COVID-19, Emergency services utilization, Peuples Autochtones, Analyse de séries chronologiques interrompues, Données de santé recueillies de façon routinière, Feux de forêt, COVID-19, Utilisation des services d’urgence

## Abstract

**Objectives:**

To explore how two major public health crises—the 2016 Fort McMurray wildfire and the COVID-19 pandemic—affected emergency care (EC) utilization among First Nations residents of the Regional Municipality of Wood Buffalo, Alberta, and to inform First Nations–led health system planning and response.

**Methods:**

We conducted a co-designed repeated cross-sectional study using interrupted time series analysis of administrative data. Monthly EC visit counts were modeled overall and for mental health and behavioural diagnoses (MBD). Two separate interrupted time series analyses were conducted, each with a single interruption point: the May 2016 wildfire (analytic window May 2014–April 2018) and April 2020 onset of COVID-19 (analytic window April 2017–March 2023). Segmented regression models (Poisson or negative binomial) estimated level and post-event slope changes stratified by age, sex, and location.

**Results:**

The wildfire was associated with geographic displacement of care. Province-wide, EC visits showed immediate level changes (+ 21.0% overall; + 43.0% MBD). Conversely, COVID-19 was associated with an immediate local decline in EC visits (− 48.2%), slope increase, and faster post-COVID growth. MBD-related visits showed stability, with no significant immediate change and slower post-pandemic growth. Crisis impacts varied by age and sex, with youth and those assigned male at birth showing the most pronounced changes.

**Conclusion:**

This study demonstrates how co-designed epidemiological methods can highlight community priorities and lived experiences through data. Repeated crises shaped EC utilization among First Nations residents in distinct ways, indicating possible disruptions in access and alterations in care-seeking patterns. Findings emphasize the importance of strengthening culturally safe, community-led, and age- and sex-responsive approaches to mental wellness, emergency preparedness, and recovery.

**Supplementary Information:**

The online version contains supplementary material available at https://doi.org/10.17269/s41997-026-01238-2.

## Résumé

**Objectifs:**

Explorer comment deux crises majeures de santé publique — le feu de forêt de Fort McMurray en 2016 et la pandémie de COVID-19 — ont influencé l’utilisation des services de soins d’urgence par les résidents des Premières Nations de la Municipalité régionale de Wood Buffalo (Alberta), afin d’éclairer la planification et les interventions des systèmes de santé dirigés par les Premières Nations.

**Méthodes:**

Nous avons réalisé une étude transversale répétée, élaborée selon une approche de coconception, en utilisant une analyse de séries chronologiques interrompues à partir de données administratives. Le nombres de visites mensuels aux services d’urgence ont été modélisés pour l’ensemble des visites ainsi que pour celles liées aux diagnostics de santé mentale et comportementaux. Deux analyses de séries chronologiques interrompues distinctes ont été effectuées, chacune comportant un point d’interruption unique: l’incendie de forêt de mai 2016 (mai 2014 à avril 2018) et le début de la pandémie de COVID-19 en avril 2020 (avril 2017 à mars 2023). Des modèles de régression segmentée (de Poisson ou binomiale négative) ont permis d’estimer les changements immédiats de niveau et les changements après l’événement, selon l’âge, le sexe et le lieu de résidence.

**Résultats:**

L’incendie de forêt a été associé à un déplacement géographique du recours aux soins. À l’échelle provinciale, les visites aux services d’urgence ont connu une augmentation immédiate (+21,0 % pour l’ensemble des visites; +43,0 % pour les visites liées à la santé mentale et comportementale). À l’inverse, la pandémie de COVID-19 a été associée à une baisse immédiate locale des visites aux services d’urgence (−48,2 %), suivie d’une augmentation graduelle et d’une croissance plus rapide après la pandémie. Les visites liées à la santé mentale et comportementale sont demeurées relativement stables, sans changement immédiat significatif et avec une croissance plus lente après la pandémie. Les effets des crises variaient selon l’âge et le sexe, les jeunes et les personnes de sexe masculin à la naissance présentant les changements les plus marqués.

**Conclusion:**

Cette étude montre comment des méthodes épidémiologiques élaborées en coconception peuvent mettre en lumière les priorités des communautés et les expériences vécues à travers les données. Les crises répétées ont influencé de manière distincte l’utilisation des services d’urgence par les résidents des Premières Nations, suggérant de possibles perturbations de l’accès aux soins et des modifications dans les comportements de recours aux services. Les résultats soulignent l’importance de renforcer des approches culturellement sécuritaires, dirigées par les communautés et adaptées à l’âge et au sexe pour favoriser le bien-être mental, la préparation aux situations d’urgence et le rétablissement.

## Introduction

In recent decades, Northern Alberta has experienced repeated public health emergencies, including the 2016 wildfire, flooding, and the COVID-19 pandemic (Sauve et al., 2024). For First Nations communities, structural conditions—rooted in colonization—shape baseline healthcare use and how crises affect utilization patterns. Jurisdictional fragmentation in healthcare responsibility, chronic underinvestment in on-reserve infrastructure, geographic isolation, and workforce instability contribute to longstanding inequities in healthcare delivery (Loppie & Wien, 2022). This creates uneven system capacity to prepare for, respond to, and recover from public health emergencies (Hahman & Kumar, 2022; Standing Committee on Indigenous & Northern Affairs, 2023).

Crises may alter healthcare utilization among First Nations by changing both healthcare service availability and health needs. The 2016 Fort McMurray (FMM) wildfire resulted in a large-scale evacuation and temporary disruption of services at the Northern Lights Regional Health Centre (NLRHC) (Sauve et al., 2024). Evidence from wildfire-affected regions documents increases in respiratory and cardiovascular morbidity related to wildfire smoke exposure, alongside elevated post-traumatic stress symptoms, depression, and substance use following displacement and loss (Montesanti et al., 2021; Thériault et al., 2021).

During the COVID-19 pandemic in Alberta, consecutive states of emergency led to suspension of non-urgent services, workforce redeployments, phased lockdowns and reopenings, visitor restrictions, and shifts toward virtual care delivery. Disruptions to routine care contributed to interruptions in chronic disease management, delayed diagnoses, and worsening social isolation (Nickel et al., 2024). As such, crises may increase demand for acute services, while simultaneously suppressing, delaying, or redirecting care-seeking due to service disruptions and access barriers.

Across Canada, routinely collected administrative data document disruptions in emergency care (EC) visits during public health crises (Canadian Institute for Health Information, 2021). EC visits provide a sensitive indicator of shifts in care utilization by capturing acute presentations when alternative services are unavailable, inaccessible, or deferred (Rose et al., 2023). However, few studies have examined crisis-related shifts in EC use among First Nations populations. Recognizing how repeated crises influence EC utilization is critical for First Nations–led health system planning and emergency preparedness. This study aimed to quantify the immediate and longer-term effects of two major public health crises on EC utilization among First Nations residents of the Regional Municipality of Wood Buffalo (RMWB), Alberta, Canada.

## Methods

### Study design

We conducted a community-partnered repeated cross-sectional study using an interrupted time series (ITS) design to explore trends, level, and slope changes in monthly EC visits associated with the 2016 FMM wildfire and the COVID-19 pandemic among First Nations residents of the RMWB. Two separate ITS analyses were conducted, each with a single interruption point: the May 2016 wildfire, using an analytic window from May 2014 to April 2018, and the April 2020 onset of COVID-19, using an analytic window from April 2017 to March 2023. Administrative health data were available from January 2014 to December 2023. Study questions, inclusion criteria, indicators, and analytic choices were co-designed with the Athabasca Tribal Council (ATC) to ensure a community-driven approach to epidemiology aligned with locally identified priorities (Hayward et al., 2021).

### Study population and data sources

We defined a population-based cohort of residents of the RMWB based on their First Nations status and postal code at encounter with healthcare services. First Nations status in administrative health data is assigned depending on inclusion in the Alberta Health Care Insurance Plan (AHCIP) First Nations Status Registry (Government of Alberta, 2024). Individuals were eligible to contribute EC visits during the study period if they had a postal code in the Canada Post Postal Code Translation File with Local Name “Wood Buffalo” recorded between April 2016 and March 2023. No minimum duration of residence or continuous residency in the RMWB was required. De-identified, person-level data were obtained from the Ministry of Primary and Preventative Health Services with deterministic linkage performed by the Health Analytics Branch prior to transfer. EC visits by eligible individuals were extracted from the Alberta Ambulatory Care Dataset, which captures all provincially funded ambulatory encounters, including visits to hospital emergency departments and urgent care centres across Alberta. Combined, these are referred to as “EC visits” throughout this manuscript since less than 1% of EC visits occurred at urgent care centres. In the RMWB, unscheduled acute care is delivered exclusively through the emergency department at the NLRHC. Dataset descriptions are available from https://open.alberta.ca/dataset/overview-of-administrative-health-datasets (accessed October 6, 2025).

### Study setting and context

The RMWB is home to approximately 3%^1^ of Alberta’s Indigenous population, one of the largest Indigenous populations in the province, with residents living on reserve, in rural communities, and in its urban centre, FMM (Statistics Canada, 2023). Vast geography, remoteness, and limited road access create persistent challenges for delivering consistent and equitable healthcare. Health services are anchored at the NLRHC and shaped by long travel distances, seasonal mobility, and a resource-based economy. This research is part of the Better Together Project, a collaborative initiative led by the ATC and Alberta Health Services (AHS) with funding from the Northern Lights Health Foundation and research support by the University of Alberta’s CARE Lab in the School of Public Health (https://www.atcfn.ca/btp). ATC represents five First Nations in the region and provides political advocacy, coordination, and service delivery support. The overall project aims to better understand healthcare utilization patterns, needs, and experiences of Indigenous Peoples in the RMWB and to advance community-driven service improvements aligned with reconciliation commitments.

### Variables

The dependent variable was the count of EC visits, measured as aggregated visit counts at the month level and analyzed overall and by diagnostic category. Population denominators were not incorporated since reliable population estimates for First Nations residents of the RMWB are unavailable. For mental health–specific analyses, visits were included if an ICD-10 F-code for Mental and Behavioural Disorders (MBD) appeared at any diagnostic position (see Online Resource 1 for included codes). This definition was guided by community partner priorities to capture all mental health-related care, including encounters in which mental health conditions were not coded as the primary diagnosis. As a sensitivity analysis, MBD-related visits were also examined using a narrower case definition restricted to visits with an MBD recorded as the primary diagnosis (see Online Resource 2).

The independent variables corresponded to two predefined events: the 2016 FMM wildfire and the onset of the COVID-19 pandemic. Interruption timing was specified a priori to avoid data-driven specification (Bernal et al., 2017), with analytic windows defined separately for each event.

For the wildfire, the interruption month was May 2016, corresponding to the FMM evacuation and the disruption of services at the NLRHC (Sauve et al., 2024). The analytic window included 24 months before and after the interruption (May 2014–April 2018), as directed by community partners. Analyses were conducted for EC visits at the NLRHC and province-wide to assess displacement of care during the evacuation.

For COVID-19, April 2020 was specified as the interruption month, representing the first full month after Alberta’s March 17 state of emergency declaration and the onset of system-level disruptions to healthcare delivery, in line with ITS guidelines (Bernal et al., 2017). The analytic window included 36 months before and after the interruption (April 2017–March 2023). Although the pandemic involved multiple waves and shifting public health measures, community partners emphasized focusing on the immediate impact and overall longer-term trajectory of EC utilization, rather than isolating the effects of individual policy phases. COVID-19 analyses were restricted to NLRHC visits to focus on local utilization patterns.

Age was categorized as youth (10–24 years) and adults (≥ 25 years), consistent with epidemiologic definitions of adolescence and young adulthood used in mental health and injury research, including frameworks that recognize ages 10–24 years as a distinct developmental and risk group (Patton et al., 2009). This also reflects community priorities to examine EC utilization within this age range as a group of particular concern.

### Statistical analysis

We modeled monthly EC visit counts using ITS segmented regression to estimate changes in EC visit volume over time. ITS models were estimated using pooled counts and stratified by age group and sex at birth (reported as male+ and female+ to acknowledge gender diversity). An overview of the model specifications, diagnostics, and sensitivity analyses (alternative interruption timing, analytic window lengths, and comparison of any-position versus primary-diagnosis MBD case definitions) is available in Online Resource 2. Models were estimated using time-segmented generalized linear models with a log link, with Poisson or negative binomial models selected based on overdispersion (*α* ≠ 0, *p* < 0.05). Influence testing (Cook’s Distance) revealed no month exceeded influence thresholds or materially altered estimates. Residual autocorrelation was evaluated using autocorrelation plots and Ljung–Box *Q*-tests. Models were estimated using Huber–White robust or Newey–West heteroskedasticity- and autocorrelation-consistent standard errors with a 12-month lag as indicated by model diagnostics.

Level and slope parameters—pre-interruption slope, immediate interruption effect/level change, change in slope (pre- vs. post-interruption trend difference), and post-interruption slope—are reported as rate ratios with 95% confidence intervals. Subgroup comparisons of regression estimates were evaluated using Wald *z*-tests. Seasonality was controlled using calendar-month fixed effects. Data management and analyses were conducted on secure, access-controlled servers (University of Alberta, Canada) using Stata/SE 19 (StataCorp LLC, College Station, TX, USA). The study is described in accordance with the REporting of studies Conducted using Observational Routinely-collected health Data (RECORD) guidelines (Benchimol et al., 2015) (Online Resource 3).

## Results

Between January 2014 and December 2023, there were 34,155 EC visits by 2810 First Nations residents of the RMWB across Alberta (mean 284.6 ± 41.6/month; Table 1). Mean visits per patient ranged from 3.8 to 4.7/month in the pre-/post-wildfire and COVID-19 observation windows. The sample was 35.8 ± 19.6 years of age and 54.7% female+. Youth accounted for 19.5% and adults for 70.2% of all EC visits. Most occurred at the NLRHC (71.0%), followed by the AHS North Zone (12.7%) and Edmonton Zone (12.6%). Mental and behavioural diagnoses (MBD) were recorded in 13.1% of visits, including 9.4% as primary diagnosis.

**Table 1:** Descriptive statistics of EC visits by First Nations residents of the RMWB across pre-/post-wildfire (24 months) and pre-/post-COVID-19 (36 months) periods. Data source: Alberta Health Ambulatory Care Dataset (Ministry of Primary and Preventative Health Services). Values shown as *n* (%) or mean (SD) as indicated

Section	Variable	Total	Pre-wildfire (24 mo)	Post-wildfire (24 mo)	Pre-COVID (36 mo)^a^	Post-COVID (36 mo)^a^
EC utilization	Total EC visits, *n*	34,155	6328	7326	8184	6989
	Monthly visits; mean (SD)	284.6 (41.6)	263.7 (24.2)	305.3 (31.6)	227.3 (30.0)	191.6 (34.0)
Study population characteristics	Unique patients; *n*	2810	1654	1790	1738	1561
	Visits per patient; mean (SD)	12.2 (17.2)	3.8 (4.9)	4.1 (5.0)	4.7 (5.9)	4.4 (7.8)
	Male+; *n* (%)	15,485 (45.3)	2754 (45.3)	3451 (47.1)	3739 (45.7)	3128 (45.3)
	Female+; *n* (%)	18,670 (54.7)	3574 (56.5)	3875 (52.9)	4445 (54.3)	3770 (54.7)
	Age (years); mean (SD)	35.8 (19.6)	34.4 (19.7)	34.6 (20.4)	33.5 (18.8)	37.8 (19.0)
	Age 10 to 24; *n* (%)	6660 (19.5)	1425 (22.5)	1506 (20.6)	1433 (17.5)	1145 (16.6)
	Age 25+; *n* (%)	23,983 (70.2)	4151 (65.6)	4861 (66.4)	5863 (71.6)	5212 (75.6)
	Urgent care visits; *n* (%)	155 (0.5)	42 (0.7)	38 (0.5)		
	NLRHC; *n* (%)	24,237 (71.0)	4462 (70.5)	4939 (67.4)	--	--
	AHS North Zone^b^; *n* (%)	4324 (12.7)	944 (14.9)	1062 (14.5)	--	--
	AHS Edmonton Zone; *n* (%)	4319 (12.6)	641 (10.1)	1072 (14.6)	--	--
	AHS Central Zone; *n* (%)	486 (1.4)	82 (1.3)	107 (1.5)	--	--
	AHS Calgary Zone; *n* (%)	512 (1.5)	100 (1.6)	101 (1.4)	--	--
	AHS South Zone; *n* (%)	277 (0.8)	99 (1.6)	45 (0.6)	--	--
Diagnosis of mental and behavioural disorder (MBD)	Any diagnostic code; *n* (%)	4489 (13.1)	571 (9.0)	853 (11.6)	923 (11.3)	1183 (16.9)
	Primary diagnosis; *n* (%)	3200 (9.4)	388 (6.1)	583 (8.0)	705 (8.6)	846 (12.1)
	Male+; *n* (%)^c^	2434 (54.2)	291 (51.0)	447 (52.4)	512 (55.5)	654 (55.3)
	Female+, *n* (%)^c^	2055 (45.8)	280 (49.0)	406 (47.6)	411 (44.5)	529 (44.7)
	Age 10 to 24; *n* (%)^c^	878 (19.6)	137 (24.0)	207 (24.3)	173 (18.7)	166 (14.0)
	Age 25+; *n* (%)^c^	3609 (80.4)	434 (76.0)	644 (75.5)	749 (81.1)	1017 (86.0)
	NLRHC; *n* (%)	3037 (67.7)	411 (72.0)	500 (58.1)	--	--

^a^Only EC visits at the NLRHC

^b^Excluding the NLRHC

^c^Among EC visits with MBD as any diagnostic code

*AHS* Alberta Health Services, *Mo* months, *NLRHC* Northern Lights Regional Health Centre (Fort McMurray). Percentages are within-period denominators unless specified

### 2016 wildfire

**Fig. 1:**
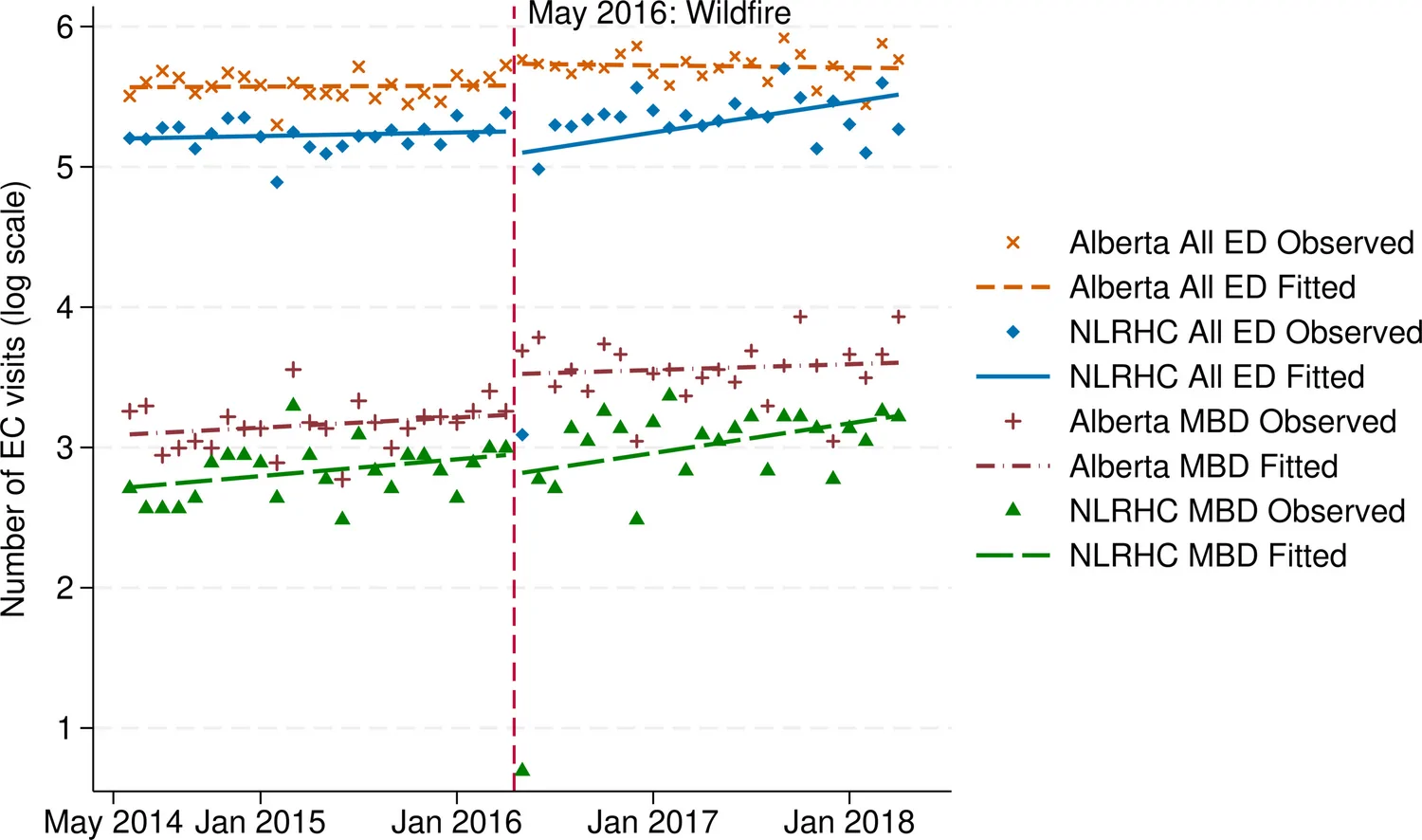
Monthly counts of EC visits surrounding the 2016 Fort McMurray wildfire (May 2014–April 2018) by First Nations residents of the RMWB showing observed and fitted values from interrupted time series analysis for all EC visits and mental health and behaviour diagnosis (MBD) visits to the local hospital (NLRHC) and across Alberta

**Fig. 2:**
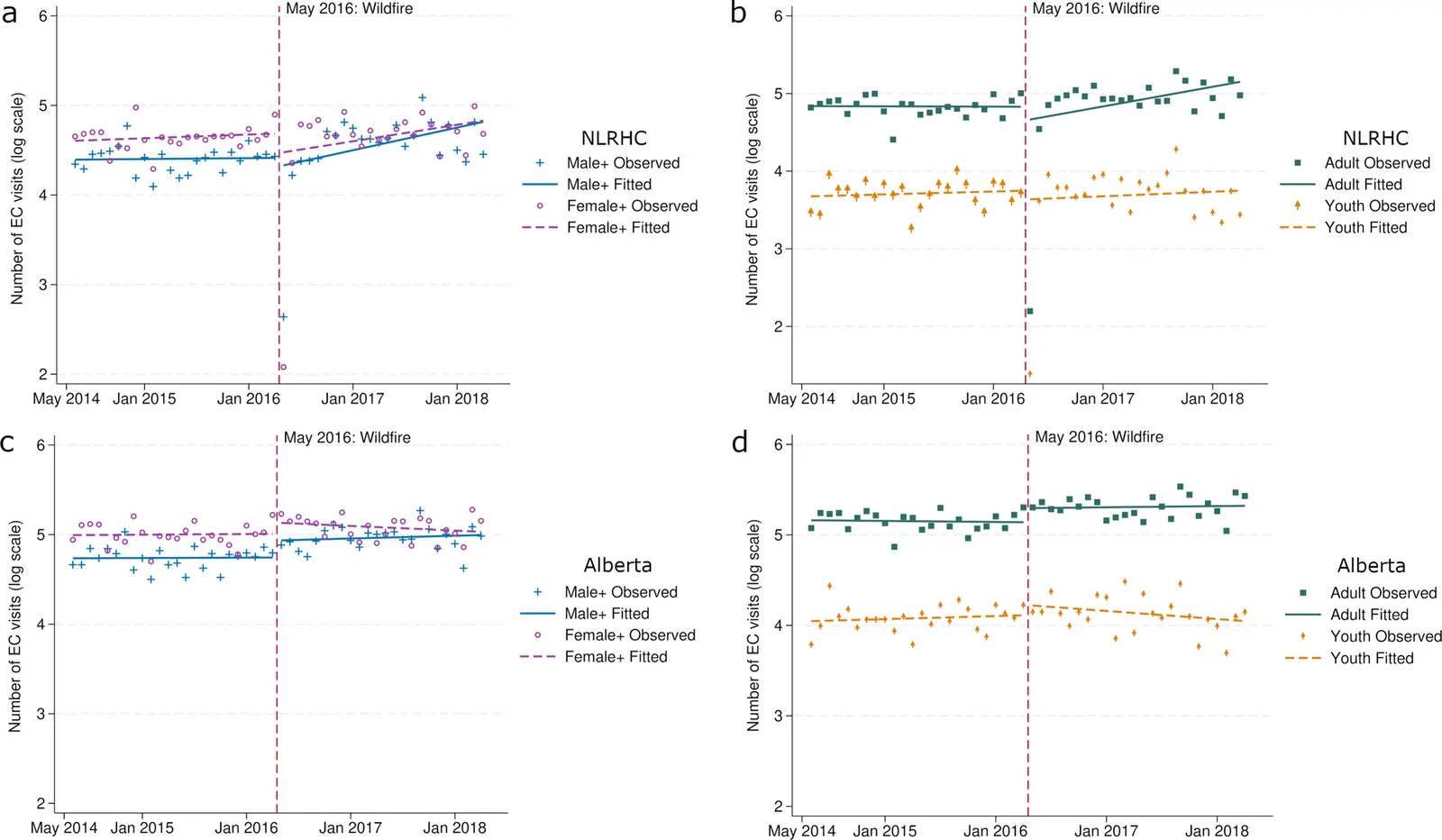
Monthly counts of overall EC visits surrounding the 2016 Fort McMurray wildfire (May 2014–April 2018) by First Nations residents of the RMWB showing observed and fitted values from interrupted time series analysis, by location, sex, and age strata: **a** EC visits at the NLRHC by sex, **b** EC visits at the NLRHC by age group, **c** EC visits across Alberta by sex, **d** EC visits across Alberta by age group

The impacts of the wildfire on EC use varied by location and outcome, with distinct geographical patterns for overall and mental health-related visits (Table 2). Table 3 presents strata comparisons. Both locally and across Alberta, monthly overall EC visits as well as sex- and age-specific slope trends were insignificant pre- and post-wildfire (Figs. 1 and 2). At the NLRHC, neither the post-wildfire immediate level nor the slope change was statistically significant.

**Table 2:** Interrupted time series estimates of monthly EC visits around the 2016 Fort McMurray wildfire (interruption: May 2016). Outcomes are shown for overall EC visits and for mental and behavioural diagnoses (MBD) visits, by sex/age, among First Nations residents of the RMWB, at the NLRHC and across Alberta, for the 2014–2018 analysis window (24 months pre- and post-wildfire). Rate ratios (RR) and 95% confidence intervals (CI) are reported for the pre-interruption trend, the immediate level change in May 2016, slope change compared to pre-wildfire, and the post-interruption trend. Statistically significant results (*p *< 0.05) are **bolded**. Data source: Alberta Health Ambulatory Care Dataset (Ministry of Primary and Preventative Health Services)

Outcome		Stratum	Pre-wildfire slope	Level change	Slope change	Post-wildfire slope
			RR (95% CI)	RR (95% CI)	RR (95% CI)	RR (95% CI)
All EC visits	NLRHC^a^	Pooled	1.000 (0.991–1.010)	0.604 (0.244–1.491)	1.016 (0.990–1.043)	1.016 (0.995–1.038)
		Male+^b^	0.998 (0.989–1.008)	0.586 (0.246–1.396)	1.020 (0.995–1.046)	1.019 (0.997–1.041)
		Female+^b^	1.002 (0.991–1.013)	0.622 (0.236–1.641)	1.012 (0.984–1.041)	1.014 (0.991–1.037)
		Youth^b^	1.005 (0.995–1.015)	0.819 (0.318–2.108)	1.002 (0.975–1.029)	1.006 (0.984–1.030)
		Adults^b^	0.997 (0.987–1.007)	0.532 (0.200–1.418)	1.021 (0.994–1.050)	1.083 (0.995–1.042)
	Alberta	Pooled^a^	1.001 (0.997–1.005)	**1.210 (1.109–1.320)**	0.998 (0.994–1.002)	0.999 (0.997–1.001)
		Male+	0.999 (0.994–1.006)	1.158 (0.932–1.438)	1.002 (0.995–1.010)	1.002 (0.996–1.008)
		Female+^a^	1.002 (0.998–1.006)	**1.245 (1.047–1.480)**	0.995 (0.988–1.002)	0.997 (0.993–1.001)
		Youth	1.005 (0.996–1.014)	1.355 (0.985–1.865)	0.990 (0.985–1.865)	0.995 (0.987–1.003)
		Adults	0.999 (0.994–1.005)	1.103 (0.927–1.313)	1.002 (0.996–1.009)	1.001 (0.997–1.006)
MBD visits	NLRHC	Pooled	1.004 (0.994–1.015)	0.824 (0.456–1.487)	1.008 (0.991–1.024)	1.012 (0.998–1.027)
		Male+^b^	1.004 (0.984–1.024)	0.933 (0.463–1.887)	1.008 (0.983–1.033)	1.011 (0.994–1.029)
		Female+^b^	1.005 (0.985–1.026)	0.717 (0.342–1.503)	1.008 (0.981–1.035)	1.012 (0.995–1.031)
		Youth^b^	**1.036 (1.008–1.065)**	1.248 (0.463–3.364)	0.978 (0.943–1.014)	1.013 (0.988–1.038)
		Adults^b^	0.997 (0.983–1.011)	0.751 (0.395–1.428)	1.015 (0.996–1.035)	1.011 (0.996–1.027)
	Alberta	Pooled	1.006 (0.996–1.015)	**1.430 (1.007–2.031)**	0.997 (0.986–1.009)	1.003 (0.994–1.013)
		Male+	**1.007 (1.000–1.013)**	**1.618 (1.292–2.027)**	0.994 (0.987–1.001)	1.001 (0.995–1.007)
		Female+	1.005 (0.988–1.022)	1.254 (0.736–2.136)	1.001 (0.979–1.023)	1.006 (0.991–1.020)
		Youth	**1.036 (1.015–1.057)**	**1.921 (1.095–3.372)**	**0.971 (0.949–0.994)**	1.006 (0.992–1.020)
		Adults	0.997 (0.984–1.010)	1.304 (0.866–1.963)	1.006 (0.991–1.021)	1.002 (0.992–1.013)

^a^Estimates from negative binomial regression. All other models were estimated using Poisson regression. For more details, see Online Resource 2, Table S2a

^b^Because evacuation and service disruption confounded immediate wildfire effects at the NLRHC, strata-specific immediate changes should be interpreted with caution

*NLRHC* Northern Lights Regional Health Centre (Fort McMurray)

**Table 3:** Interrupted time series subgroup comparisons of monthly EC visits by First Nations residents of the RMWB around the 2016 Fort McMurray wildfire (interruption: May 2016). Differences are shown by location, i.e., between NLRHC and Alberta, and across sex and age strata, for both overall EC visits and mental and behavioural diagnoses (MBD) visits among First Nations residents of the RMWB, over the 2014–2018 analysis window (24 months pre- and post-wildfire). *Z*-values and corresponding *p*-values are reported for the pre-interruption slope, the immediate level change in May 2016, the slope change compared to pre-COVID, and the post-interruption slope. Statistically significant results (*p *< 0.05) are **bolded**. Data source: Alberta Health Ambulatory Care Dataset (Ministry of Primary and Preventative Health Services)

Outcome		Comparison	Pre-wildfire slope *z*-value (*p*)	Level change *z*-value (*p*)	Slope change *z*-value (*p*)	Post-wildfire slope *z*-value (*p*)
All EC visits		NLRHC vs. Alberta	−0.133 (*p *= 0.894)	−1.500 (*p *= 0.134)	1.319 (0.187)	1.188 (*p *= 0.235)
	NLRHC	Male+ vs. female+	−0.488 (*p *= 0.626)	−0.090 (*p *= 0.928)	0.438 (*p *= 0.661)	0.235 (*p *= 0.814)
		Youth vs. adults	1.125 (*p *= 0.261)	−0.282 (*p *= 0.778)	0.438 (*p *= 0.661)	−0.548 (*p *= 0.584)
	Alberta	Male+ vs. female+	−0.518 (*p *= 0.604)	−0.512 (*p *= 0.609)	1.398 (*p *= 0.162)	0.850 (*p *= 0.395)
		Youth vs. adults	1.096 (*p *= 0.273)	1.642 (*p *= 0.101)	−1.838 (*p *= 0.066)	−0.756 (*p *= 0.450)
MBD visits		NLRHC vs. Alberta	−0.189 (*p *= 0.850)	−1.573 (*p *= 0.116)	0.989 (*p *= 0.323)	0.708 (*p *= 0.479)
	NLRHC	Male+ vs. female+	−0.079 (*p *= 0.937)	0.507 (*p *= 0.612)	0.006 (*p *= 0.995)	−0.044 (*p *= 0.965)
		Youth vs. adults	**2.491 (*p *= 0.013)**	0.373 (*p *= 0.709)	−1.789 (*p *= 0.074)	0.057 (*p *= 0.955)
	Alberta	Male+ vs. female+	0.188 (*p *= 0.851)	0.865 (*p *= 0.387)	−0.563 (*p *= 0.574)	−0.320 (*p *= 0.749)
		Youth vs. adults	**3.131 (*p *= 0.002)**	1.850 (*p *= 0.064)	−**2.526 (*p *= 0.012)**	0.200 (*p *= 0.841)

*NLRHC* Northern Lights Regional Health Centre (Fort McMurray)

**Fig. 3:**
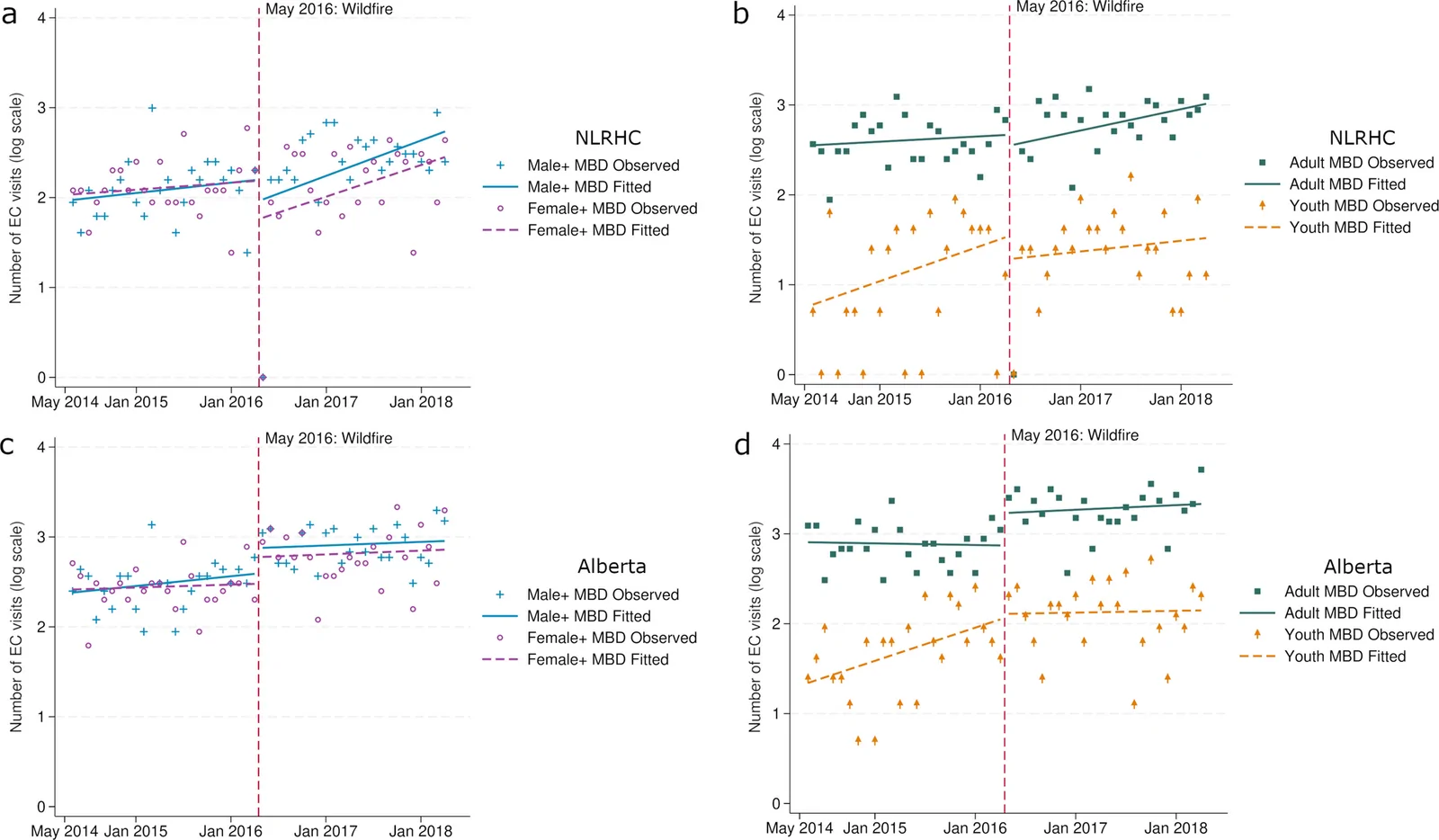
Monthly counts of mental health and behaviour (MBD)–related EC visits surrounding the 2016 Fort McMurray wildfire (May 2014–April 2018) by First Nations residents of the RMWB showing observed and fitted values from interrupted time series analysis, by location, sex, and age strata: **a** MBD visits at the NLRHC by sex, **b** MBD visits at the NLRHC by age group, **c** MBD visits across Alberta by sex, **d** MBD visits across Alberta by age group

Across Alberta, overall EC visits displayed an immediate level increase of 21.0% (*p* < 0.001) following the wildfire, with no significant slope change (Fig. 1). A similar immediate level change in EC visits was observed for female+ patients (+ 24.5%, *p* = 0.013; Fig. 2c). For MBD visits at the NLRHC, no significant pre-wildfire trend nor any short- or longer-term wildfire effects were observed (Fig. 1) with findings similar across sex strata (Fig. 3a). Pre-wildfire, youth MBD visits were increasing significantly by 3.6% per month or approximately 53.3% annually (*p* = 0.010), while adult visits were stable (Fig. 3b), resulting in a pre-wildfire slope difference between youth and adults (*z *= 2.491, *p *= 0.013). In contrast to the NLRHC, Alberta-wide MBD-related visits across Alberta jumped sharply at the interruption (+ 43.0% level change, *p *= 0.046; Fig. 1). A large immediate level change of + 61.8% (*p *< 0.001) was also observed for male+ MBD visits across Alberta (Fig. 3c). Alberta-wide youth MBD visits also increased faster than adult MBD visits before the wildfire (trend + 3.6%/month, + 52.5%/year, *p *= 0.001 vs. *p *> 0.05; *z *= 3.131, *p *= 0.002) and showed an immediate and sharp level change (+ 92%, *p *= 0.023), whereas adult MBD visits did not change significantly (Fig. 3d). Youth experienced a significant post-wildfire slope change of −2.9% per month (*p *= 0.023; Fig. 3d), whereas adult MBD visit trends remained unchanged. This slope change differed significantly between youth and adults (*z *=  − 2.523, *p *= 0.012).

### COVID-19 pandemic

**Fig. 4:**
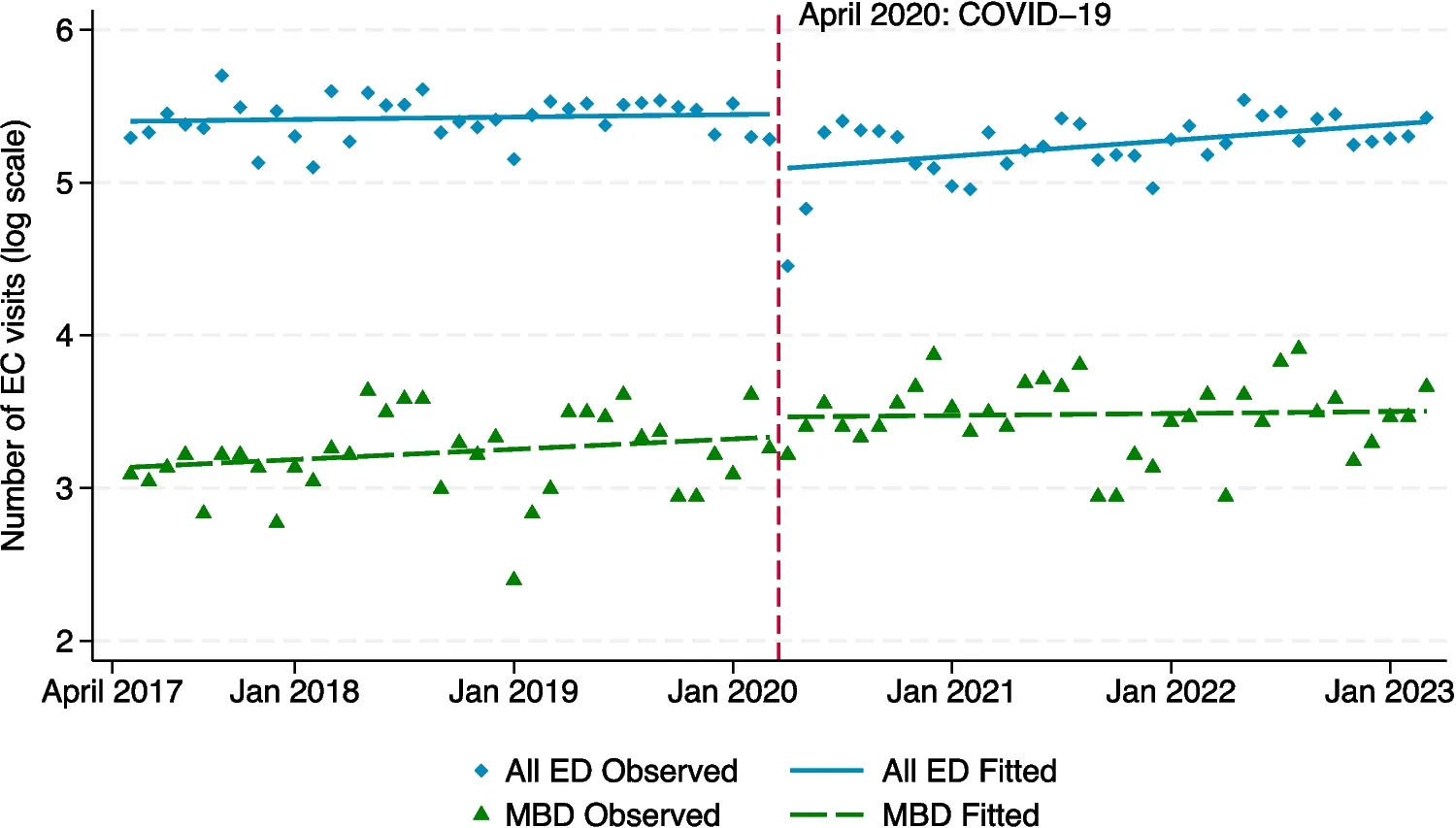
Monthly counts of EC visits to the NLRHC surrounding the start of the COVID-19 pandemic (April 2017–March 2023) by First Nations residents of the RMWB showing observed and fitted values from interrupted time series analysis for all EC visits and MBD-related visits

Changes in visit patterns associated with the onset of the COVID-19 pandemic at the NLRHC are summarized in Table 4, with estimates for subgroup comparisons in Table 5. Pooled monthly EC visits increased slightly pre-COVID (trend + 0.2%/month, + 2.7%/year, *p *= 0.042), followed by a sharp immediate level drop of 48.2% at the April 2020 interruption (*p *< 0.001) and a significant slope change (+ 0.7%/month, *p *= 0.002; Fig. 4). This resulted in a post-COVID trend with + 1.0%/month growth (+ 12.2%/year; *p *< 0.001).

**Table 4:** Interrupted time series estimates of monthly EC visit counts at the NLRHC around the COVID-19 interruption (April 2020). Outcomes are shown for overall EC visits and for mental and behavioural diagnoses (MBD) visits, by sex/age, among First Nations residents of the RMWB (analysis window: April 2017–March 2023; 36 months pre- and post-COVID). Rate ratios (RR) and 95% confidence intervals (CI) are reported for the pre-interruption trend, the immediate level change in April 2020, the slope change compared to pre-COVID, and the post-interruption slope. Statistically significant results (*p *< 0.05) are **bolded**. Data source: Alberta Health Ambulatory Care Dataset (Ministry of Primary and Preventative Health Services)

Outcome	Stratum	Pre-COVID slope	Level change	Slope change	Post-COVID slope
		RR (95% CI)	RR (95% CI)	RR (95% CI)	RR (95% CI)
All EC visits^a^	Pooled	**1.002 (1.000–1.004)**	**0.518 (0.414–0.647)**	**1.007 (1.003–1.012)**	**1.010 (1.006–1.013)**
	Male+	0.999 (0.996–1.001)	**0.560 (0.455–0.689)**	**1.008 (1.004–1.013)**	**1.007 (1.003–1.010)**
	Female+	**1.005 (1.001–1.010)**	**0.481 (0.346–0.669)**	1.007 (0.999–1.014)	**1.012 (1.007–1.018)**
	Youth	**0.994 (0.989–0.999)**	**0.372 (0.272–0.507)**	**1.018 (1.009–1.027)**	**1.012 (1.007–1.017)**
	Adults	**1.006 (1.002–1.009)**	**0.655 (0.510–0.841)**	1.002 (0.996–1.008)	**1.007 (1.004–1.011)**
MBD visits	Pooled	**1.008 (1.003–1.012)**	1.256 (0.836–1.888)	0.995 (0.987–1.004)	1.003 (0.997–1.009)
	Male+	**1.013 (1.009–1.017)**	1.486 (0.952–2.320)	**0.989 (0.981–0.997)**	1.002 (0.994–1.009)
	Female+	1.001 (0.992–1.010)	1.025 (0.638–1.648)	1.003 (0.992–1.015)	1.005 (0.997–1.012)
	Youth	0.994 (0.981–1.009)	1.801 (0.754–4.304)	0.992 (0.972–1.012)	0.987 (0.971–1.003)
	Adults	**1.011(1.005–1.016)**	1.214 (0.780–1.890)	0.995 (0.986–1.004)	1.006 (0.999–1.013)

^a^Estimates from negative binomial regression. MBD visit models were estimated using Poisson regression. For more details, see Online Resource 2, Table S2a

**Table 5:** Interrupted time series subgroup comparisons of monthly EC visits by First Nations residents of the RMWB around the COVID-19 interruption (April 2020). Differences are shown across sex and age strata, for both overall EC visits and mental and behavioural diagnoses (MBD) visits among First Nations residents of the RMWB, over the April 2017–March 2023 analysis window (36 months pre- and post-interruption). *Z*-values and corresponding *p*-values are reported for the pre-interruption slope, the immediate level change in April 2020, the slope change compared to pre-COVID, and the post-interruption slope. Statistically significant results (*p *< 0.05) are **bolded**. Data source: Alberta Health Ambulatory Care Dataset (Ministry of Primary and Preventative Health Services)

Outcome	Comparison	Pre-COVID slope *z*-value (*p*)	Level change *z*-value (*p*)	Slope change *z*-value (*p*)	Post-COVID slope *z*-value
**All EC visits**	Male+ vs. female+	**−3.810 (*p *< 0.001)**	1.530 (*p *= 0.126)	0.535 (*p *= 0.593)	**−2.500 (*p *= 0.012)**
	Youth vs. adults	**−7.071 (*p *< 0.001)**	**−10.800 (*p *< 0.001)**	**6.903 (*p *< 0.001)**	0.199 (*p *= 0.444)
**MBD visits**	Male+ vs. female+	**2.941 (*p *= 0.003)**	**2.572 (*p *= 0.010)**	**−4.293 (*p *< 0.001)**	0.300 (*p *= 0.764)
	Youth vs. adults	**−4.935 (*p *< 0.001)**	**3.343 (*p *< 0.001)**	−0.901 (*p *= 0.367)	−1.061 (*p *= 0.289)

**Fig. 5:**
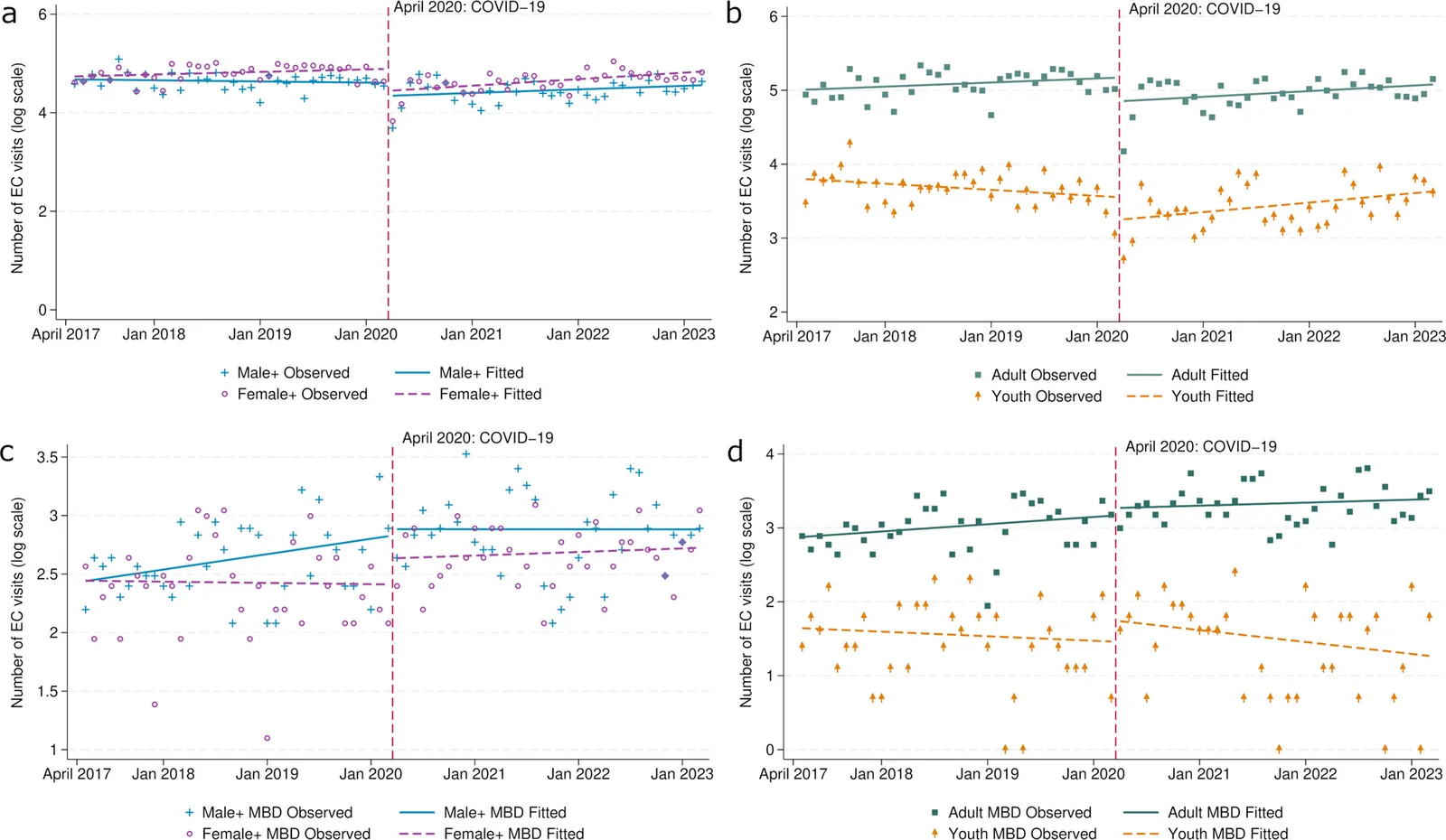
Monthly counts of EC visits to the NLRHC surrounding the start of the COVID-19 pandemic (April 2017–March 2023) by First Nations residents of the RMWB showing observed and fitted values from interrupted time series analysis, by diagnosis, sex, and age strata: **a** all EC visits by sex, **b** all EC visits by age group, **c** MBD visits by sex, **d** MBD visits by age group

Across sex strata, female+ EC visits increased significantly more sharply compared to male+ visits, both pre-COVID (trend + 0.5%/month, + 6.7%/year, *p *= 0.023 vs. *p *> 0.05; *z *= −3.810, *p *< 0.001) and post-COVID (+ 1.2%/month, + 15.7%/year vs. + 0.7%/month, + 8.4%/year both *p *< 0.001; *z *= −2.500, *p *= 0.012; Fig. 5a). Both male+ and female+ EC visits had a significant immediate level drop (−44.0% vs. −51.9%, *p *< 0.001). The slope change was only significantly different for male+ EC visits (+ 0.8%/month; *p *< 0.001), and no significant between-group differences were observed in the level or slope change.

Age-stratified models also indicated significant pre-COVID differences with an opposite trend (−0.6%/month, −6.6%/year, *p *= 0.026 for youth vs. + 0.6%/month, + 7.1%/year, *p *= 0.001 for adults; *z *= −7.071, *p *< 0.001), and a much larger immediate level decrease for youth (−62.8%, *p *< 0.001 vs. −34.5% in adults, *p *= 0.001; *z *= −10.800, *p *< 0.001; Fig. 5b). Post-COVID, youth visits showed a reversal of the slope direction (+ 1.8%/month slope change, *p *< 0.001), while no significant slope change was observed among adults, indicating a significant difference in slope change between age groups (*z *= 6.903, *p *< 0.001). Post-COVID slopes showed increasing EC visit counts for both youth (+ 1.2%/month or ~ 15.4%/year, *p *< 0.001) and adults (+ 0.7%/month or ~ 9.4%/year, *p *< 0.001).

Pooled MBD-related visits showed increasing visit counts before COVID-19 (trend + 0.8%/month, + 9.4%/year, *p *= 0.002), with no significant immediate level or slope changes (Fig. 4). Visits by males + showed fast pre-COVID growth (+ 1.3%/month, + 16.4%/year, *p *< 0.001), no significant immediate level change, and a stable trend post-COVID due to a decline in growth rate (slope change: −1.1%/month, *p *= 0.009; Fig. 5c). In contrast, female+ MBD visits showed no significant pre-COVID trend or interruption-related changes, resulting in significant sex differences, except for the post-COVID slope (Table 5).

Adult MBD visits showed pre-COVID growth (+ 1.1%/month, + 13.4%/year, *p *< 0.001), while youth visits showed no significant pre-COVID trend, revealing age-specific pre-COVID slopes (*z *= −4.935, *p *< 0.001; Fig. 5d). No significant COVID-related immediate level or slope changes were observed for either stratum, even though the immediate level change difference between youth and adults was statistically significant (*z *= 3.343, *p *< 0.001). Sensitivity analyses restricting MBD-related EC visits to those with MBD as the primary diagnosis differed from the primary model (see Online Resource 2). The primary-diagnosis model showed a significant immediate increase of 46.3% (*p *< 0.001) and a post-COVID trend of 0.7% per month or approximately 8.9% per year (*p *= 0.006).

## Discussion

Two major crises—the 2016 FMM wildfire and the COVID-19 pandemic—disrupted EC use by First Nations residents of the RMWB in distinct ways, demonstrating how impacts were shaped by care location, visit type, and population group. The May 2016 wildfire produced immediate and geographically differentiated shifts in EC use as a result of the FMM evacuation and disruptions to local service provision, including a 2-week closure of the NLRHC. Across Alberta, EC visits increased at the interruption month, consistent with displaced RMWB residents seeking care elsewhere. MBD-related visits showed a similar pattern, with an immediate level increase observed province-wide, especially among youth and those born male.

Qualitative research supports these findings, documenting emotional distress and depressive symptoms among Indigenous residents of the RMWB following the disaster (Montesanti et al., 2021). Other research has shown that mental health impacts of wildfires—including post-traumatic stress disorder, depression, anxiety, and insomnia—can persist for years after the event (To et al., 2021). However, we did not observe a sustained increase in overall or MBD-related EC use following the wildfire, suggesting that longer-term impacts were not reflected in EC utilization patterns, rather than absent. EC data capture only a subset of clinical healthcare encounters and cannot distinguish whether observed patterns reflect increased need, unmet need, or shifts to alternative forms of care, such as care accessed through community-based pathways not captured in administrative data (Dodd et al., 2018). Additionally, the mobilization and expansion of mental health services and supports—including short-term, community-based supports, e.g., time-limited counselling delivered intermittently in communities—during the recovery period may have influenced patterns of EC use (Fitzpatrick et al., 2021). Shifts in service availability may have reduced reliance on EC or redirected care, contributing to the absence of sustained post-wildfire increases in MBD-related EC visits. Future research could explicitly model the acute evacuation period separately from longer-term recovery and account for evolving service availability to better distinguish displacement effects from sustained mental health impacts.

COVID-19 produced an abrupt disruption. At the NLRHC, overall EC visits decreased by nearly half at the onset of the pandemic, similar to provincial evidence showing reductions in emergency department use among both First Nations and non-First Nations populations with diabetes in Alberta (Weaver et al., 2025). The primary, community-directed research question focused on the broader spectrum of MBD-related EC utilization, including visits where mental health concerns were recorded in any diagnostic position. In this primary model, MBD-related EC visits had been increasing before the pandemic and did not show a significant immediate level change at COVID-19 onset, in contrast to the sharp suppression in overall EC utilization. This suggests that broader MBD-related EC utilization persisted during the pandemic period rather than declining. However, sensitivity analyses using a narrower primary-diagnosis definition showed a significant immediate increase by 46.3% at COVID-19 onset, followed by a significant post-COVID increase. This indicates that visits with MBD as primary reason for care may have increased at pandemic onset and continued to grow over time, even though broader MBD-related EC utilization remained comparatively stable. Studies in non-Indigenous populations showed that MBD visits first declined, then increased more rapidly in post-pandemic years (Chadi et al., 2021; Overhage et al., 2023). Our findings align with national evidence of worsening mental health among First Nations, Inuit, and Métis early in the pandemic, including increased stress, anxiety, and depression due to disruptions in culturally safe services and community supports (Arriagada et al., 2020; Hahman & Kumar, 2022).

Youth mental health emerged as particularly affected. Before the wildfire, youth experienced the steepest increases in MBD-related visits, which subsequently slowed. During COVID-19, youth showed a sharper immediate decline and faster recovery than adults in overall EC visits. Together, these findings suggest youth EC utilization may be particularly responsive to system disruptions and changes in access. Nonetheless, these patterns cannot determine whether underlying mental health burden changed or shifted to other forms of care, because administrative data do not capture community-based or culturally grounded care. Accordingly, EC utilization should be interpreted as one dimension of healthcare use rather than a comprehensive measure of youth well-being, lived experience, or service access.

Importantly, EC records include only individuals who are documented within the provincial health system, which does not include on-reserve healthcare (Webb, 2022). To this point, fewer than half of the estimated First Nations population in the RMWB had recorded EC visits during the study period (Statistics Canada, 2023). Moreover, many disaster impacts—including stress, grief, displacement, cultural disruption, and community trauma—may not translate into EC visits. Anecdotal evidence indicates that Indigenous Elders avoided accessing the hospital during COVID-19. Furthermore, Indigenous communities took their own public health measures, further limiting travel and access independent of provincial measures (Banning, 2020; Webb, 2022). Consequently, the absence of strong immediate or sustained statistical signals in EC data should not be interpreted as absence of impact or need. Rather, it reflects limitations of clinical and EC-based data in capturing complex health, social, and cultural effects, and potential redistribution of care across fragmented and time-limited services and supports. This underscores that statistically non-significant findings may still represent meaningful, important impacts at the community level.

### Public health implications

Findings from this study highlight the need to strengthen and pre-position alternative care pathways to reduce pressure on EC services during emergencies (Venugopal et al., 2021), while anticipating spillover effects and ensuring surge capacity in neighbouring regions. Emergency and pandemic preparedness, response, and recovery must recognize that already strained EC services may be limited in their ability to provide appropriate mental healthcare, given constraints on time, continuity, cultural safety, and therapeutic environment (Levin & Aburub, 2024; Marchand et al., 2024). Investment in non-EC mental health pathways—particularly culturally rooted, land- and community-based services—remains critical (Venugopal et al., 2021). Health-system planning should also prioritize First Nations youth, people assigned male at birth, and others disproportionately affected, reflecting distinct patterns of care use across acute response and long-term recovery.

Overall, the literature on emergency and pandemic preparedness, response, and recovery in Indigenous contexts emphasizes the importance of Indigenous-led—or co-designed/co-governed—planning and service delivery (Halseth & Murdock, 2020; Montesanti et al., 2019). Emerging scholarship and policy guidance call for the braiding of Indigenous and Western knowledge (Indigenous Services Canada, 2024; Wieman & Malhotra, 2023), resourcing Indigenous patient navigation and crisis teams (Marchand et al., 2024; Rankin et al., 2022), and ensuring Indigenous data sovereignty to support equity, effectiveness, accountability, and learning (Nickel et al., 2024). In the RMWB, the 2019 Indigenous Mental Health Forum underscored the need for services grounded in Indigenous languages, traditions, and support networks, yet stakeholders still report that investments in First Nations–driven initiatives—e.g., trauma teams, wellness centres, and treatment facilities—remain insufficient to meet community needs (Montesanti et al., 2022).

### Strengths and limitations

To our knowledge, this is the first study in Alberta—and among the first on Turtle Island (North America)—to use an ITS design to explore the impacts of public health emergencies on health service utilization among First Nations, a population known to experience substantial barriers to care (McLane et al., 2021). A key strength is its co-development with First Nations partners to ensure it reflects locally identified priorities and advances a community-driven approach to epidemiology. A distinguishing feature of this study is its regional analytic focus. By examining EC utilization within a specific health service context rather than provincial-level aggregation, we were able to detect localized, context-dependent dynamics—e.g., care displacement during the wildfire evacuation and site-specific declines during COVID-19—often obscured in broader analyses. These findings underscore the importance of regionally grounded analyses in public health to inform locally relevant, First Nations–led planning and response.

Several limitations should be noted. First, administrative health data, including EC data, may underestimate service use and only capture sex at birth, limiting gender-based analyses (Boulanger et al., 2025). Cohort identification relied on provincial health data and AHCIP First Nations status records, which undercount First Nations residents (e.g., non-status or incomplete registrations) and may misclassify residence due to lagged address updates, introducing potential selection and misclassification bias. The absence of comparator groups limited our ability to distinguish First Nations–specific effects from broader regional or system-level influences.

Second, we modeled monthly visit counts without a population denominator given the lack of reliable time-varying First Nations population estimates (Snyder et al., 2024). Therefore, findings reflect shifts in service volume rather than per-capita utilization, and thus may partly capture shifts in population size, composition, or mobility rather than in healthcare utilization alone.

Third, the youth category of 10–24 years combines younger adolescents, older adolescents, and young adults. Although this grouping was chosen a priori based on epidemiologic definitions and community priorities, it may mask developmental and service-use differences within this age range. Given that youth showed pronounced changes in several analyses, findings should be interpreted as broad patterns among people aged 10–24 rather than as applying uniformly across all youth.

Finally, ITS assumes no concurrent events at the interruption point. However, overlapping factors—including changes in service availability during the post-wildfire recovery period and the April 2020 flood in FMM during COVID-19—represent important concurrent influences, limiting causal attribution of observed changes to each interruption alone. While this study identifies broad patterns, future research could utilize more granular ITS approaches to further elucidate phase-specific dynamics. Taken together, these limitations suggest our findings should be interpreted cautiously and complemented with community-based and qualitative evidence to generate a fuller picture of First Nations’ health needs and experiences during crises.

## Conclusion

This study demonstrates how epidemiological research methods co-designed with First Nations partners can generate data-driven insights grounded in community priorities and lived experiences. Using an ITS approach, we traced broad patterns in how EC use among First Nations residents of the RMWB changed during two crises in distinct ways. Findings highlight opportunities to strengthen culturally safe care, mental wellness supports, emergency preparedness, and disaster recovery through age- and sex-specific approaches, while also underscoring important gaps in what administrative data can capture about health and lived experience. Through the Better Together Project, these data support First Nations–led service development, informing future investments and showcasing how epidemiology can serve as a tool for storytelling, accountability, and advancing self-determined health futures.

## Contributions to knowledge

What does this study add to the existing knowledge?

- First interrupted time series analysis of wildfire and COVID-19 impacts on First Nations emergency care use in Alberta and Turtle Island (North America).
- Suggests wildfire was associated with geographic displacement of care, while COVID-19 was associated with reduced overall emergency care but stable mental health-related visits.
- Identifies heterogeneity: youth and people assigned male at birth showed the greatest changes in emergency care use during crises.
- Demonstrates co-developed, community-driven approach to epidemiology aligned with First Nations priorities.

What are the key implications for public health interventions, practice, or policy?

- Strengthen Indigenous-led emergency and pandemic preparedness, response, and recovery.
- Invest in culturally grounded, community-based mental healthcare pathways beyond emergency care.
- Resource Indigenous patient navigation and mobile crisis teams across emergency care and community-based settings.
- Prioritize high-need groups—particularly youth and people assigned male at birth—for targeted mental wellness supports.
- Uphold Indigenous data sovereignty through community-driven monitoring and evaluation that drives equity, effectiveness, accountability, and learning.

## Supplementary Information

Below is the link to the electronic supplementary material.

ESM 1 (PDF 289 KB)

ESM 2 (PDF 314 KB)

ESM 3 (PDF 193 KB)

## Data Availability

The administrative health datasets used in this study are held by the Ministry of Primary and Preventative Health Services and Alberta Health Services. The ATC governed use of First Nations’ data in accordance with OCAP® principles. Data access requests require approval from ATC and the provincial custodians; please contact the corresponding author for guidance.

## Footnotes

1 Likely underestimated given the incomplete enumeration in the Census of Population of certain Indigenous reserves and settlements (Statistics Canada, 2023).
