# Supplementary material for: Patterns of emergency care utilization among First Nations residents of Wood Buffalo, Alberta, during public health crises: An interrupted time series analysis
Source: Can J Public Health. 2026 Jul 23;117(5):852–66. doi: 10.17269/s41997-026-01238-2 (PMC13680587; doi:10.17269/s41997-026-01238-2)
Supplement: Supplementary file 1 [file 41997_2026_1238_MOESM1_ESM.pdf]

Online Resource 1: ICD-10 Code List for Mental & Behavioural Diagnoses

Mental and behavioural disorder (MBD)–related EC visits were identified using ICD-10 Chapter V (F00–F99) codes recorded in any diagnostic position. Codes F70–F89 (Intellectual disabilities and Disorders of psychological development) were excluded, as we report on emergency care (EC) visits for mental health and substance use, which focus on acute presentations. Exclusion of these codes improves interpretability of interrupted time-series estimates by restricting the outcome to conditions expected to vary with acute system disruptions.

Table S1. ICD-10 Chapter V (F00–F99) codes included in MBD–related emergency care (EC) visit analyses among First Nations residents of the Regional Municipality of Wood Buffalo (RMWB). MBD–related EC visits were identified using ICD-10 codes from Chapter V (F00–F99: Mental and Behavioural Disorders) recorded in any diagnostic position in the Alberta Ambulatory Care Database between January 2014 and December 2023. All subcategory codes within each block mentioned (e.g., F32.0–F32.9) were included. Codes were grouped according to standard ICD-10 Chapter V classifications. **Source:** World Health Organization (2019). International Classification of Diseases and Related Health Problems 10th revision. Geneva, Switzerland: WHO. https://www.who.int/standards/classifications/classification-of-diseases. Accessed 14 Apr 2025.

ICD-10 Code Range	Diagnostic Category	Description
F00–F09	Organic mental disorders	Dementia, delirium, and other organic or symptomatic mental disorders
F10–F19	Substance-related disorders	Mental and behavioural disorders due to psychoactive substance use (alcohol, opioids, cannabis, stimulants, etc.)
F20–F29	Schizophrenia and psychotic disorders	Schizophrenia, schizotypal disorder, delusional disorders, acute psychosis
F30–F39	Mood (affective) disorders	Manic episodes, bipolar disorder, depressive episodes, recurrent depression
F40–F48	Anxiety and stress-related disorders	Phobic anxiety, generalized anxiety, OCD, PTSD, adjustment disorders, somatoform disorders
F50–F59	Behavioural syndromes associated with physiological disturbances	Eating disorders, nonorganic sleep disorders, sexual dysfunction
F60–F69	Personality and adult behavioural disorders	Personality disorders, impulse disorders, gender identity disorders
F90–F98	Childhood and adolescent behavioural disorders	ADHD, conduct disorders, emotional disorders of childhood
F99	Unspecified mental disorder	Mental disorder, not otherwise specified
