# Supplementary material for: Patterns of emergency care utilization among First Nations residents of Wood Buffalo, Alberta, during public health crises: An interrupted time series analysis
Source: Can J Public Health. 2026 Jul 23;117(5):852–66. doi: 10.17269/s41997-026-01238-2 (PMC13680587; doi:10.17269/s41997-026-01238-2)
Supplement: Supplementary file 2 [file 41997_2026_1238_MOESM2_ESM.pdf]

Online Resource 2: Expansion of Methods: Interruption points, analysis windows, model diagnostics, and sensitivity analyses for interrupted time-series models

Interruption points and analysis windows

This study evaluated two predefined interruption events: the May 2016 Fort McMurray wildfire and the April 2020 onset of the COVID-19 pandemic. Each interruption was modeled using segmented regression terms for: (1) continuous time (pre-interruption trend); (2) a binary indicator for the interruption month (immediate level change); (3) an interaction between time and the post-interruption indicator (change in slope) and (4) the post-interruption trend.

Interruption timing points were specified *a priori* to avoid data-driven specification (Bernal et al., 2017), with analytic windows defined separately for each event. For the wildfire analysis, the interruption month was May 2016, corresponding to the evacuation of Fort McMurray and disruption of services at the NLRHC (Public Safety Canada, 2016). The analytic window included 24 months before and after the interruption (May 2014–April 2018). Although administrative data were available beginning January 2014, a symmetric 24-month pre- and post-interruption window was intentionally selected to preserve balance around the interruption and support stable estimation of level and slope parameters in segmented regression. Simulation studies indicate that interrupted time-series models perform more reliably when sufficient pre- and post-interruption observations are available to estimate trends and autocorrelation structures (Turner et al., 2021). Extending the post-interruption period to two years was a community-identified priority, reflecting explicit interest from First Nations leadership in evaluating not only the immediate disruption associated with the wildfire but also sustained changes in EC utilization over time. Analyses were conducted for EC visits at the NLRHC and province-wide to assess potential displacement of care during evacuation.

The interruption point for the COVID-19 analyses was specified as April 2020, representing the first full month following Alberta's March 17 state of emergency declaration and the onset of system-level disruptions to healthcare delivery. In interrupted time-series analyses, interventions occurring mid-interval are commonly specified at the first full time unit after implementation to align the interruption point with the temporal unit of the data (Bernal et al., 2017). The analytic window included 36 months before and after the interruption (April 2017–March 2023) to provide sufficient observations to estimate baseline trends and seasonality and to assess sustained changes in level and slope over the course of a prolonged public health emergency, with the last state of emergency in Alberta ending in February 2022. Although the pandemic involved multiple waves and shifting public health measures, community partners emphasized that the priority was not to isolate the effects of each discrete policy phase, but rather to understand the immediate structural shift at pandemic onset and the longer-term trajectory of EC utilization over time. COVID-19 analyses were restricted to NLRHC visits to focus on local utilization patterns.

Statistical analysis: Model diagnostics and sensitivity analyses for interrupted time-series models

We modeled monthly EC visit counts using interrupted time series (ITS) segmented regression to estimate changes in EC visit volume over time. ITS models were estimated using pooled counts and stratified by age group (youth 10–24; adults ≥25) and sex at birth (reported as male+ and female+ to acknowledge gender diversity). Models were estimated using time-segmented generalized linear models with a log link. Poisson regression was specified, with overdispersion evaluated using likelihood ratio tests comparing Poisson and negative binomial models ($\alpha = 0$); negative binomial models were retained when α differed significantly from zero ($p < 0.05$). Model diagnostics also included influence testing using Cook's distance ($D > 1$; Table S2a). No month exceeded influence thresholds or materially altered level or slope estimates.

Residual autocorrelation was evaluated using autocorrelation (ACF) plots of deviance residuals and Ljung–Box Q-tests for serial dependence (Ljung & Box, 1978)(Table S2b). Residual diagnostics did not indicate meaningful serial correlation if autocorrelations were within 95% confidence bounds and Ljung–Box tests were not statistically significant. Such models were estimated using heteroskedasticity-consistent (Huber–White) robust standard errors to account for potential heteroskedasticity without specifying an autocorrelation structure (Huber, 1967; White, 1980). In some models, especially COVID-19 models, a spike exceeding the 95% confidence bounds was observed at lag 12 in the ACF across model specifications with a statistically significant Ljung–Box test, indicating annual dependence in the monthly series. Those models were estimated using Newey–West heteroskedasticity- and autocorrelation-consistent standard errors with a 12-month lag to account for seasonal autocorrelation (Newey & West, 1986). Additional sensitivity analyses included alternative interruption timing (e.g., shifting the COVID-19 interruption month (Table S2c)) and analytic window lengths for both the 2016 wildfire and COVID-19 models (Table S2d).

We also conducted a sensitivity analysis comparing MBD case definitions for both wildfire and COVID-19 models (Table S2e). The primary analysis included EC visits with an MBD code recorded in any diagnostic position, consistent with the community-directed aim of capturing the broader spectrum of mental health-related EC utilization. The sensitivity analysis restricted MBD visits to those with MBD recorded as the primary diagnosis to examine visits where MBD was documented as the primary reason for care. Findings were substantively similar for the wildfire analysis, while COVID-19 findings differed at the immediate level change and post-COVID slope. These differences are reported in the Results and interpreted in the Discussion of the manuscript. Across alternative timing and analytic-window specifications, effect estimates and inferences were substantively unchanged; however, the MBD case-definition sensitivity analysis indicated that COVID-19 findings differed when MBD was restricted to the primary diagnosis, as reported in the manuscript.

Section 1. Model Diagnostics

Table S2a. Summary of dispersion and influence diagnostics for interrupted time-series analyses of monthly emergency care (EC) visits among First Nations residents of the Regional Municipality of Wood Buffalo (RMWB). Diagnostics are presented for wildfire models at the Northern Lights Regional Health Centre (NLRHC) and all other Alberta facilities (excluding NLRHC), and for COVID-19 models at the NLRHC. Outcomes include overall EC visits and mental and behavioural diagnosis (MBD)–related visits. Overdispersion was assessed using the likelihood-ratio test of α=0 (p<0.05) and influence at the month level using Cook's distance, with values greater than 1 (D > 1) considered indicative of potentially influential observations. **Data source:** Alberta Health Ambulatory Care Dataset (Ministry of Primary and Preventative Health Services).

Analysis			Outcome	Overdispersion (LR test α = 0, p-value)	Maximum Cook's D (Month)	Influential Month (D > 1)?	Regression Model Used
May 2016 Wildfire	All EC Visits	NLRHC	Pooled	0.0373 (p < 0.001)	0.0370 (25)	No	Negative Binomial
			Male+	0.0293 (p < 0.001)	0.0309 (25)	No	Negative Binomial
			Female+	0.0334 (p < 0.001)	0.0419 (25)	No	Negative Binomial
			Youth	0.0111 (p = 0.047)	0.0383 (25)	No	Negative Binomial
			Adult	0.0374 (p < 0.001)	0.0424 (25)	No	Negative Binomial
		Alberta	Pooled	0.0027 (p = 0.001)	0.0015 (22)	No	Negative Binomial
			Male+	0.0020 (p = 0.113)	0.3374 (43)	No	Poisson
			Female+	0.0034 (p = 0.013)	0.0027 (20)	No	Negative Binomial
			Youth	0.0023 (p = 0.247)	0.2764 (29)	No	Poisson
			Adult	0.0017 (p = 0.076)	0.2479 (41)	No	Poisson
	MBD Visits	NLRHC	Pooled	5.63e-12 (p = 1.000)	0.4478 (25)	No	Poisson
			Male+	7.83e-08 (p = 0.500)	0.3201 (25)	No	Poisson
			Female+	2.64e-08 (p = 0.500)	0.2941 (23)	No	Poisson
			Youth	1.19e-25 (p = 1.000)	0.2358 (23)	No	Poisson
			Adult	4.45e-08 (p = 0.500)	0.3397 (25)	No	Poisson
		Alberta	Pooled	2.59e-08 (p =0.500)	0.1958 (48)	No	Poisson
			Male+	1.34e-08 (p = 0.499)	0.1542 (35)	No	Poisson
			Female+	2.78e-08 (p = 0.500)	0.1936 (48)	No	Poisson
			Youth	2.91e-08 (p = 0.500)	0.0878 (44)	No	Poisson
			Adult	4.45e-08 (p = 0.500)	0.2705 (35)	No	Poisson
COVID-19	All EC Visits	NLRHC	Pooled	0.0101 (p < 0.001)	0.0041 (37)	No	Negative Binomial
			Male+	0.0121 (p < 0.001)	0.0034 (37)	No	Negative Binomial
			Female+	0.0086 (p < 0.001)	0.0046 (37)	No	Negative Binomial
			Youth	0.0157 (p = 0.002)	0.0046 (36)	No	Negative Binomial
			Adult	0.0122 (p < 0.001)	0.0048 (37)	No	Negative Binomial
	MBD Visits	NLRHC	Pooled	0.0080 (p = 0.100)	0.0078 (45)	No	Poisson
			Male+	0.0118 (p = 0.126)	0.0132 (45)	No	Poisson
			Female+	0.0032 (p = 0.403)	0.0099 (14)	No	Poisson
			Youth	0.0357 (p = 0.178)	0.0157(70)	No	Poisson
			Adult	0.0106 (p = 0.077)	0.0084 (45)	No	Poisson

Table S2b. Residual autocorrelation diagnostics for interrupted time-series models of monthly EC visits among First Nations residents of the RMWB. Residual autocorrelation up to 12 lags was assessed using correlograms and autocorrelation (ACF) plots of deviance residuals and Ljung–Box Q-tests for serial dependence. Models were estimated using Huber–White robust standard errors or Newey–West heteroskedasticity- and autocorrelation-consistent (HAC) standard errors with a 12-month lag (Huber, 1967, Newey & West, 1986, White, 1980) in the event of significant autocorrelation (significant Ljung-Box Q-test). Wildfire models were evaluated at the NLRHC and for all other Alberta facilities (excluding NLRHC). COVID-19 models were evaluated at the NLRHC. All models include calendar-month fixed effects. Statistically significant results (p<0.05) are **bolded**. **Data source:** Alberta Health Ambulatory Care Dataset (Ministry of Primary and Preventative Health Services).

Analysis			Outcome	ACF Notable Lags[1]	Maximum |r| (Lag)	Ljung–Box Q (lag=12)[2]	(Ljung–Box p-value)	Final Variance Specification Selected
May 2016 Wildfire	All EC Visits	NLRHC	Pooled	No	0.2873 (12)	7.0285	0.8557	Robust (Huber–White)
			Male+	No	0.2864 (12)	9.4043	0.6681	Robust (Huber–White)
			Female+	No	0.2658 (12)	9.5551	0.6549	Robust (Huber–White)
			Youth	Yes	0.3700 (12)	13.9033	0.3069	Robust (Huber–White)
			Adult	No	0.2647 (12)	7.1218	0.8495	Robust (Huber–White)
		Alberta	Pooled	Yes	0.3248 (12)	22.3567	**0.0337**	Newey–West HAC, lag 12
			Male+	Yes	0.3229 (5)	10.8809	0.5392	Robust (Huber–White)
			Female+	Yes	0.4578 (12)	32.9284	**0.0010**	Newey–West HAC, lag 12
			Youth	Yes	0.3805 (12)	13.4313	0.3385	Robust (Huber–White)
			Adult	No	0.2997 (12)	14.1499	0.2912	Robust (Huber–White)
May 2016 Wildfire	MBD Visits	NLRHC	Pooled	None	0.2803 (12)	12.1201	0.4361	Robust (Huber–White)
			Male+	Yes	0.3362 (12)	12.4865	0.4074	Robust (Huber–White)
			Female+	None	0.2953 (12)	17.4252	0.1343	Robust (Huber–White)
			Youth	Yes	0.3501 (1)	20.6750	0.0553	Robust (Huber–White)
			Adult	None	0.2698 (9)	20.2377	0.0627	Robust (Huber–White)
		Alberta	Pooled	None	0.2386 (10)	13.0581	0.3648	Robust (Huber–White)
			Male+	Yes	0.3495 (9)	28.3915	**0.0048**	Newey–West HAC, lag 12
			Female+	None	0.3014 (9)	16.4605	0.1710	Robust (Huber-White)
			Youth	None	0.2254 (12)	13.6467	0.3238	Robust (Huber-White)
			Adult	Yes	0.3493 (10)	17.8253	0.1211	Robust (Huber-White)
COVID-19	All EC Visits	NLRHC	Pooled	Yes	0.3203 (12)	27.9923	**0.0055**	Newey–West HAC, lag 12
			Male+	Yes	0.2959 (12)	31.3549	**0.0017**	Newey–West HAC, lag 12
			Female+	Yes	0.2770 (12)	15.1438	0.2337	Robust (Huber-White)
			Youth	Yes	0.3539 (1)	22.6778	**0.0306**	Newey–West HAC, lag 12
			Adult	Yes	0.3433 (12)	21.6404	**0.0418**	Newey–West HAC, lag 12
	MBD Visits	NLRHC	Pooled	Yes	0.2795 (12)	28.2574	**0.0051**	Newey-West HAC, lag 12
			Male+	Yes	0.3771 (12)	38.3953	**0.0001**	Newey-West HAC, lag 12
			Female+	Yes	0.2676 (5)	16.9574	0.1512	Robust (Huber-White)
			Youth	No	0.2464 (10)	17.6341	0.1273	Robust (Huber-White)
			Adult	Yes	0.5653 (1)	22.5799	**0.0315**	Newey-West HAC, lag 12

[1] Notable lag if falling outside Bartlett confidence bounds.

[2] The Ljung–Box Q-test evaluates whether residual autocorrelations up to a specified lag *h* are jointly zero. We evaluated the test up to lag 12, corresponding to one annual seasonal cycle in the monthly data (Brockwell & Davis, 2022).

Notes: ACF: Autocorrelation function; HAC: heteroskedasticity- and autocorrelation consistent.

Section 2. Sensitivity Analyses

Supplemental Table S2c. Sensitivity analysis of interrupted time-series estimates of monthly EC visits among First Nations residents of the RMWB at the NLRHC around the onset of the COVID-19 pandemic, using alternative interruption points. The primary model specifies April 2020 as the interruption point, with sensitivity analyses specifying March 2020 and May 2020 as alternative interruption months. Models include calendar-month fixed effects. Rate Ratios (RR) and 95% Confidence Intervals (CI) are reported for the pre-interruption slope, the immediate level change at interruption, the slope change compared to pre-interruption, and the post-interruption slope. All models include calendar-month fixed effects. Statistically significant results ($p<0.05$) are **bolded**. **Data source:** Alberta Health Ambulatory Care Dataset (Ministry of Primary and Preventative Health Services).

Parameter	Primary Model<br>April 2020 Interruption<br>RR (95% CI)	Sensitivity Analysis<br>March 2020 Interruption<br>RR (95% CI)	Sensitivity Analysis<br>May 2020 Interruption<br>RR (95% CI)
Pre-interruption slope	**1.002 (1.000-1.004)**	**1.004 (1.002-1.005)**	1.000 (0.994–1.005)
Immediate level change	**0.518 (0.414-0.647)**	**0.542 (0.458-0.641)**	**0.548 (0.468–0.641)**
Slope change	**1.007 (1.003-1.012)**	**1.005 (1.002-1.009)**	**1.009 (1.003–1.015)**
Post-interruption slope	**1.009 (1.006-1.013)**	**1.009 (1.007-1.012)**	**1.008 (1.006-1.010)**

Supplemental Table S2d. Sensitivity analysis of interrupted time-series estimates of monthly EC visits among First Nations residents of the RMWB at the NLRHC using alternative analytic time windows. The primary models use full analytic windows (24 months before and after May 2016 for the wildfire analysis and 36 months before and after April 2020 for the COVID-19 analysis), with sensitivity analyses restricting the observation period to shorter windows (12 months before and after May 2016 and 24 months before and after April 2020). Rate Ratios (RR) and 95% Confidence Intervals (CI) are reported for the pre-interruption slope, the immediate level change at interruption, the slope change compared to pre-interruption, and the post-interruption slope. All models include calendar-month fixed effects and the same variance specification as the primary analysis. Statistically significant results ($p<0.05$) are **bolded**. **Data source:** Alberta Health Ambulatory Care Dataset (Ministry of Primary and Preventative Health Services).

Parameter	Primary Model<br>Full analytic window<br>RR (95% CI)	Sensitivity Analysis<br>Shorter analytic window<br>RR (95% CI)
**Wildfire (May 2016 Interruption)**	**24 / 24 months**	**12 / 12 months**
Pre-interruption slope	1.000 (0.991–1.010)	0.970 (0.741-1.269)
Immediate level change	0.604 (0.244-1.491)	0.338 (0.010-10.955)
Slope change	1.016 (0.990-1.043)	1.078 (0.974-1.193)
Post-interruption slope	1.016 (0.994-1.038)	1.045 (0.798-1.368)
**COVID-19 (April 2020 Interruption)**	**36 / 36 months**	**24 / 24 months**
Pre-interruption slope	**1.002 (1.000-1.004)**	1.000 (0.995-1.005)
Immediate level change	**0.518 (0.414-0.647)**	**0.555 (0.409-0.754)**
Slope change	**1.007 (1.002-1.012)**	1.009 (0.999-1.019)
Post-interruption slope	**1.009 (1.006-1.013)**	**1.009 (1.002-1.016)**

Supplemental Table S2e. Sensitivity analysis of interrupted time series estimates of monthly mental and behavioural disorder (MBD) EC visits by First Nations residents of the RMWB to the NLRHC around the COVID-19 interruption (analysis window: April 2017–March 2023; 36 months pre- and post-COVID) using alternative case definitions. The primary models define MBD visits as those with *any* diagnosis of a mental or behavioural disorder, with sensitivity analyses restricting the outcome to visits with a primary diagnosis of MBD (ICD-10 F-codes). Models include calendar-month fixed effects. Rate Ratios (RR) and 95% Confidence Intervals (CI) are reported for the pre-interruption trend, the immediate level change in April 2020, the slope change compared to pre-COVID, and the post-interruption slope. Statistically significant results ($p<0.05$) are **bolded**. **Data sources:** Alberta Health Ambulatory Care Dataset (Ministry of Primary and Preventative Health Services).

	**Primary Model**	**Sensitivity Analysis**
**Parameter**	**Any diagnosis of MBD** RR (95% CI)	**Primary diagnosis of MBD** RR (95% CI)
**Wildfire (May 2016 Interruption)**		
Pre-interruption slope	1.004 (0.994-1.015)	1.012 (0.995-1.028)
Immediate level change	0.824 (0.456-1.487)	1.260 (0.660-2.407)
Slope change	1.008 (0.991-1.024)	0.993 (0.973-1.014)
Post-interruption slope	1.012 (0.998-1.027)	1.005 (0.988-1.021)
**COVID-19 (April 2020 Interruption)**		
Pre-COVID slope	**1.008 (1.003-1.012)**	**1.012 (1.005-1.020)**
Immediate level change	1.256 (0.836-1.888)	**1.463 (1.114-1.920)**
Slope change	0.995 (0.981-0.997)	0.995 (0.987-1.003)
Post-COVID slope	1.003 (0.997-1.009)	**1.007 (1.004-1.010)**

References

Bernal, J. L., Cummins, S., & Gasparrini, A. (2017). Interrupted time series regression for the evaluation of public health interventions: a tutorial. *International journal of epidemiology*, 46(1), 348-355. https://doi.org/10.1093/ije/dyw098

Brockwell, P. J., & Davis, R. A. (Eds.). (2022). *Introduction to time series and forecasting*. New York, NY: Springer New York.

Huber, P. J. (1967). The behavior of maximum likelihood estimates under nonstandard conditions. In L. M. Le Cam & J. Neyman (Eds.), *Fifth Berkeley Symposium on Mathematical Statistics and Probability. Volume 1: Statistics* (Vol. 5.1, pp. 221-233). Berkeley, CA: University of California Press.

Newey, W. K., & West, K. D. (1986). A simple, positive semi-definite, heteroskedasticity and autocorrelationconsistent covariance matrix. *Econometrica*, 55(3), 703-708. https://doi.org/10.2307/1913610

Turner, S. L., Forbes, A. B., Karahalios, A., Taljaard, M., & McKenzie, J. E. (2021). Evaluation of statistical methods used in the analysis of interrupted time series studies: a simulation study. *BMC Med Res Methodol*, 21(1), 181. https://doi.org/10.1186/s12874-021-01364-0

White, H. (1980). A Heteroskedasticity-Consistent Covariance Matrix Estimator and a Direct Test for Heteroskedasticity. *Econometrica*, 48(4), 817-838. https://doi.org/10.2307/1912934
